# Supplementary material for: The entorhinal-DG/CA3 pathway in the medial temporal lobe retains visual working memory of a simple surface feature
Source: eLife. 2023 Mar 2;12:e83365. doi: 10.7554/eLife.83365 (PMC10019891; doi:10.7554/eLife.83365)
Supplement: Supplementary file 1. — (A) Tests of statistical significance in the neural similarity across trials captured by the similarity of the cued item. (B) Tests of statistical significance in IEM results for the cued item. (C) The number of voxels included for each bilateral ROI in each subject. [file elife-83365-supp1.docx]

**Supplementary File 1**

***Supplementary File 1a***. Tests of Statistical Significance in the Neural Similarity across Trials Captured by the Similarity of the Cued item

|  | t | df | Cohen's d | p-uncorrected | p-Bonferroni |
| --- | --- | --- | --- | --- | --- |
| aLEC | 4.29 | 15 | 1.11 | 0.0006 | **0.0052** |
| pMEC | 2.16 | 15 | 0.56 | 0.047 | n.s. |
| Perirhinal | 3.00 | 15 | 0.78 | 0.0089 | n.s. |
| Para-hippocampus | 2.70 | 15 | 0.70 | 0.017 | n.s. |
| DG/CA3 | 3.65 | 15 | 0.94 | 0.0024 | **0.019** |
| CA1 | 1.56 | 15 | 0.40 | 0.14 | n.s. |
| Subiculum | 2.53 | 15 | 0.65 | 0.023 | n.s. |
| Amygdala | 0.06 | 15 | 0.02 | 0.95 | n.s. |

*Note*: n.s. = non-significant. It is well-acknowledged that multiple comparisons would inflate TYPE-I error, and hence we adopted a relatively conservative correction procedure (Bonferroni correction, see Rosenthal and Rubin, 1983 for some discussion). This procedure reveals the significance test outcomes that could not be attributed to chance alone. Yet, it does not imply that the non-significant results indicate no effect. Rather, as shown in the table, non-significant effects are often associated with an attenuated effect size in the same direction, indicating unstable estimate across participants. We therefore focus on the regions where robust effects have been identified across participants after the correction of multiple comparisons (i.e., aLEC and DG/CA3).

***Supplementary File 1b***. Tests of Statistical Significance in IEM results for the Cued item

|  | t | df | Cohen's d | p-uncorrected | p-Bonferroni |
| --- | --- | --- | --- | --- | --- |
| aLEC | 4.41 | 15 | 1.14 | 0.00050 | **0.0041** |
| pMEC | 2.30 | 15 | 0.59 | 0.036 | n.s. |
| Perirhinal | 1.97 | 15 | 0.51 | 0.067 | n.s. |
| Para-hippocampus | 1.46 | 15 | 0.38 | 0.17 | n.s. |
| DG/CA3 | 4.73 | 15 | 1.22 | 0.00030 | **0.0021** |
| CA1 | 1.09 | 15 | 0.28 | 0.29 | n.s. |
| Subiculum | 1.78 | 15 | 0.46 | 0.10 | n.s. |
| Amygdala | -0.13 | 15 | -0.03 | 0.90 | n.s. |

*Note*: n.s. = non-significant. It is well-acknowledged that multiple comparisons would inflate TYPE-I error, and hence we adopted a relatively conservative correction procedure (Bonferroni correction, see Rosenthal and Rubin, 1983 for some discussion). This procedure reveals the significance of test outcomes that could not be attributed to chance alone. Yet, it does not imply that the non-significant results indicate no effect. Rather, as shown in the table, non-significant effects are often associated with an attenuated effect size in the same direction, indicating an unstable estimate across participants. We therefore focus on the regions where robust effects have been identified across participants after the correction of multiple comparisons (i.e., aLEC and DG/CA3).

***Supplementary File 1c.*** The number of voxels included for each bilateral ROI in each subject

| Subject ID | Entorhinal | | Perirhinal | Para-hippocampus | Hippocampus | | | Amygdala |
| --- | --- | --- | --- | --- | --- | --- | --- | --- |
|  | aLEC | pMEC |  |  | DG/CA3 | CA1 | Subiculum |  |
| 1 | 269 | 167 | 504 | 1350 | 379 | 1006 | 416 | 858 |
| 2 | 253 | 120 | 688 | 1094 | 302 | 625 | 300 | 800 |
| 3 | 220 | 175 | 564 | 1250 | 359 | 875 | 339 | 754 |
| 4 | 308 | 126 | 774 | 1104 | 355 | 859 | 410 | 865 |
| 5 | 308 | 162 | 805 | 1293 | 347 | 884 | 331 | 852 |
| 6 | 248 | 124 | 907 | 1343 | 340 | 881 | 385 | 914 |
| 7 | 218 | 111 | 634 | 1071 | 295 | 729 | 288 | 801 |
| 8 | 205 | 142 | 388 | 940 | 304 | 732 | 301 | 874 |
| 9 | 254 | 134 | 846 | 1372 | 297 | 734 | 314 | 712 |
| 10 | 230 | 136 | 412 | 1118 | 294 | 770 | 422 | 882 |
| 11 | 273 | 147 | 884 | 1296 | 362 | 880 | 342 | 851 |
| 12 | 245 | 127 | 714 | 1119 | 314 | 818 | 296 | 782 |
| 13 | 257 | 169 | 833 | 1326 | 288 | 879 | 430 | 1044 |
| 14 | 219 | 120 | 920 | 1261 | 328 | 771 | 257 | 753 |
| 15 | 225 | 152 | 649 | 1418 | 326 | 845 | 413 | 879 |
| 16 | 211 | 105 | 431 | 1199 | 262 | 679 | 255 | 640 |
| Mean | 246.44 | 138.56 | 684.56 | 1222.13 | 322.00 | 810.44 | 343.69 | 828.81 |
| s.e.m. | 7.90 | 5.39 | 45.35 | 33.55 | 8.16 | 24.10 | 15.18 | 23.15 |

Note: s.e.m. = standard error, which is the standard deviation divided by the square root of sample size.
